# RNA m^6^A reader YTHDF2 facilitates lung adenocarcinoma cell proliferation and metastasis by targeting the AXIN1/Wnt/β-catenin signaling

**DOI:** 10.1038/s41419-021-03763-z

**Published:** 2021-05-13

**Authors:** Yin Li, Hao Sheng, Feng Ma, Qiang Wu, Jianfang Huang, Qiang Chen, Lianghe Sheng, Xinghai Zhu, Xiaoxi Zhu, Meng Xu

**Affiliations:** 1 Department of Oncology, The First Affiliated Hospital of Jinan University, Jinan University, Guangzhou, P. R. China; 2 National Cancer Center/National Clinical Research Center for Cancer/Cancer Hospital & Shenzhen Hospital, Chinese Academy of Medical Sciences and Peking Union Medical College, Shenzhen, P. R. China; 3 Department of Medical Biochemistry and Molecular Biology, School of Medicine, Jinan University, Guangzhou, Guangdong China; 4 Department of Biotechnology, College of Life Science and Technology, Jinan University, Guangzhou, P. R. China

**Keywords:** DNA methylation, Non-small-cell lung cancer

## Abstract

Lung adenocarcinoma (LUAD) remains a leading cause of cancer-related deaths worldwide. YTHDF2 is a reader of N^6^-methyladenosine (m^6^A) on RNA and plays a critical role in the initiation and propagation of myeloid leukemia; however, whether YTHDF2 controls the development of LUAD remains to be explored. Here, we found that YTHDF2 was significantly upregulated in LUAD compared with paracancerous normal tissues, and YTHDF2 knockdown drastically inhibited, while its overexpression promoted, cell growth, colony formation and migration of LUAD cells in vitro. In addition, YTHDF2 knockdown significantly inhibited tumorigenesis in a murine tumor xenograft model. Through the integrative analysis of RNA-seq, m^6^A-seq, CLIP-seq, and RIP-seq datasets, we identified a set of potential direct targets of YTHDF2 in LUAD, among which we confirmed AXIN1, which encodes a negative regulator of the Wnt/β-catenin signaling, as a direct target of YTHDF2. YTHDF2 promoted AXIN1 mRNA decay and subsequently activated the Wnt/β-catenin signaling. Knockout of AXIN1 sufficiently rescued the inhibitory effect of YTHDF2 depletion on lung cancer cell proliferation, colony-formation, and migration. These results revealed YTHDF2 to be a contributor of LUAD development acting through the upregulation of the AXIN1/Wnt/β-catenin signaling, which can be a potential therapeutic target for LUAD.

## Introduction

Lung cancer is the leading cause of cancer-related mortality globally^1^. Lung adenocarcinoma (LUAD) represents the most common subtype, accounting for ∼40%, of lung cancer. LUAD patients are generally diagnosed late in the course of the disease^2^. Despite of the advancement of molecular diagnostics, targeted therapies and immunotherapies, the average 5-year survival rate of LUAD remains as low as 15%^3,4^. Therefore, understanding the molecular mechanism of the initiation and progression of LUAD is essential for developing future diagnostic and therapeutic strategies.

Epigenetic modifications are deeply involved in oncology. The N6-methyladenosine (m6A) modification of RNA is a reversible and dynamic epigenetic event other than DNA and histone modifications^5–7^. m6A is mostly found on mRNA in eukaryotic cells, but also exists on lncRNA, circRNA, microRNA, tRNA, rRNA, and snoRNA. N6-methylation is usually found in the adenosine embedded in the consensus sequence RRm6ACH, especially near the stop codon of mRNA transcripts. mRNA destiny and functions, such as alternative polyadenylation and pre-mRNA splicing, mRNA stability, decay, localization, transport, and translation, are regulated by proteins that catalyze the formation, removal, and reading of m6A^8–11^. The functions of these proteins in cancer are not completely understood. METTL3 and METL14, two m6A methyltransferases, exert completely opposite effects on the migration of hepatocellular carcinoma^12,13^. METTL3 facilitates cell growth, survival and invasion in gastric cancer, but plays an antioncogene role and is a favorable prognostic factor in colorectal cancer^14–16^. YTH N6-methyladenosine RNA binding protein 2 (YTHDF2) is a member of the YTH domain family^17,18^. It has been reported that YTHDF2 recognizes dynamic m6A modification to influence the translation status and lifespan of mRNA^8,19^. By contrast, Sheng demonstrated that YTHDF2 directly binds to the m6A modification site of the three prime untranslated region (3′-UTR) of 6-phosphogluconate dehydrogenase (6PGD) to promote 6PGD mRNA translation, but does not cause transcription degradation^20^. YTHDF2 promoted tumorigenesis of acute myeloid leukemia (AML) through recognizing m6A on, and promoting degradation of, TNFR2 mRNA, resulting in reduced sensitivity of AML Cells to TNF^21^. YTHDF2 inhibits cell growth and proliferation by destroying the EGFR mRNA in hepatocellular carcinoma, but promotes cell growth by promoting 6-phosphogluconate dehydrogenase mRNA translation in lung cancer^20,22^. Therefore, YTHDF2 may exert different effects on different cancers by targeting mRNA transcripts of different genes, and further elucidation of the role of YTHDF2 and its new targets in the development of LUAD is needed.

In our present study, we showed that YTHDF2 expression was abnormally up-regulated in LUAD, and that YTHDF2 was crucial for lung cancer cell tumorigenesis and metastasis. We also identified a new target of YTHDF2, AXIN1, which is a negative regulator of Wnt/β-catenin pathway. Knockdown of YTHDF2 promoted expression of AXIN1, leading to the inhibition of the Wnt/β-catenin pathway. In summary, our data revealed YTHDF2-catenin AXIN1-Wnt/β-catenin to be a new pathway favoring LUAD tumorigenesis and metastasis, which can be targeted for the treatment LUAD.

## Results

### YTHDF2 was highly expressed in lung adenocarcinoma

**Fig. 1:**
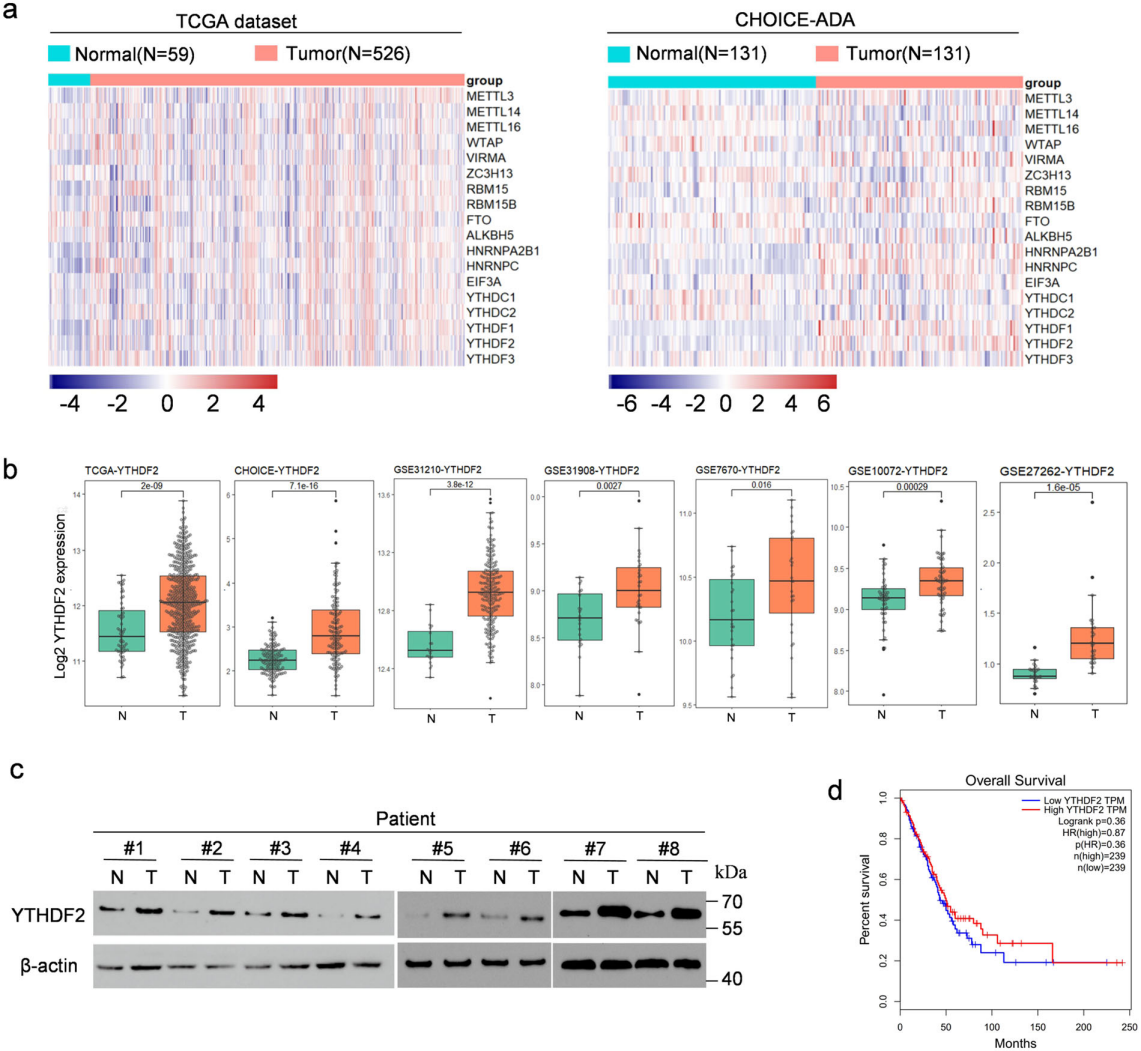
YTHDF2 was highly expressed in LUAD. **a** Heat map profiling the expression of m6A WERs in TCGA (left) and CHOICE (right) databases of LUAD. **b** Relative RNA levels of YTHDF2 in LUAD tissue and normal lung tissue in TCGA, CHOICE, and GEO datasets. **c** Immunoblotting assay of YTHDF2 expression in eight paired LUAD primary tumor samples. β-actin was used as a loading control. **d**) Kaplan–Meier analysis of LUAD cancer in TCGA for the correlations between YTHDF2 expression and overall survival.

We analyzed The Cancer Genome Atlas (TCGA) and CHOICE datasets of Chinese patients with NSCLC to evaluate the expression profiles of m6A “write”, “eraser”, and “reader” genes (WERs) in LUAD^23^. The results showed that in both datasets, the mRNA expression levels of METTL3, VIRMA, RBM15, RBM15B, HNRNPA2B1, HNRNPC, EIF3A, YTHDF1, and YTHDF2 were significantly increased, and FTO in LUAD was significantly decreased compared with adjacent normal tissue. Interestingly, YTHDC1 and YTHDC2 showed opposite trends in TCGA and CHOICE; whereas, the other WERs showed no significant difference in both or one of the databases (Fig. 1a and Fig. s1a). In general, YTHDF2 was highly expressed in most human cancers from TCGA pan-cancer database; whereas, its mRNA expression in KICH, KIRP, PCPG, THCA, and THYM was notably lower than that in adjacent normal tissue (Fig. s1b). The clinical characteristics of the TCGA and CHOICE samples were summarized in Additional file 1: Tables S1 and S2. The differences in the expression profiles of YTHDF2 prompted us to investigate its functional characteristics and clinical outcomes in LUAD. Then, we analyzed the expression of YTHDF2 in LUAD based on five GEO datasets (GSE31210, GSE31908, GSE7670, GSE10072, and GSE27262), and found that YTHDF2 in LUAD was upregulated compared with that in normal lung epithelium cells (Fig. 1b)^24–27^. We detected the expression of YTHDF2 protein in eight LUAD and paired adjacent normal lung tissues through western blot analysis to further validate the expression level of YTHDF2. The results revealed that YTHDF2 protein expression level in LUAD tissue was higher than that in adjacent tissue (Fig. 1c).

The further analysis of TCGA survival data revealed that higher YTHDF2 expression was not correlated with better overall survival (Fig. 1d). We then analyzed the association between YTHDF2 expression and LUAD prognosis in GEO datasets. In line with previous findings from the TCGA database, our results further verified that higher YTHDF2 expression did not indicate improved overall survival in patients with LUAD (Fig. s1c). Taken together, these data suggested that the YTHDF2 mRNA was not a prognostic marker of the overall survival of patients with LUAD, but the higher YTHDF2 protein level was closely associated with LUAD.

### YTHDF2 levels regulated LUAD cell proliferation and viability in vitro and in vivo

**Fig. 2:**
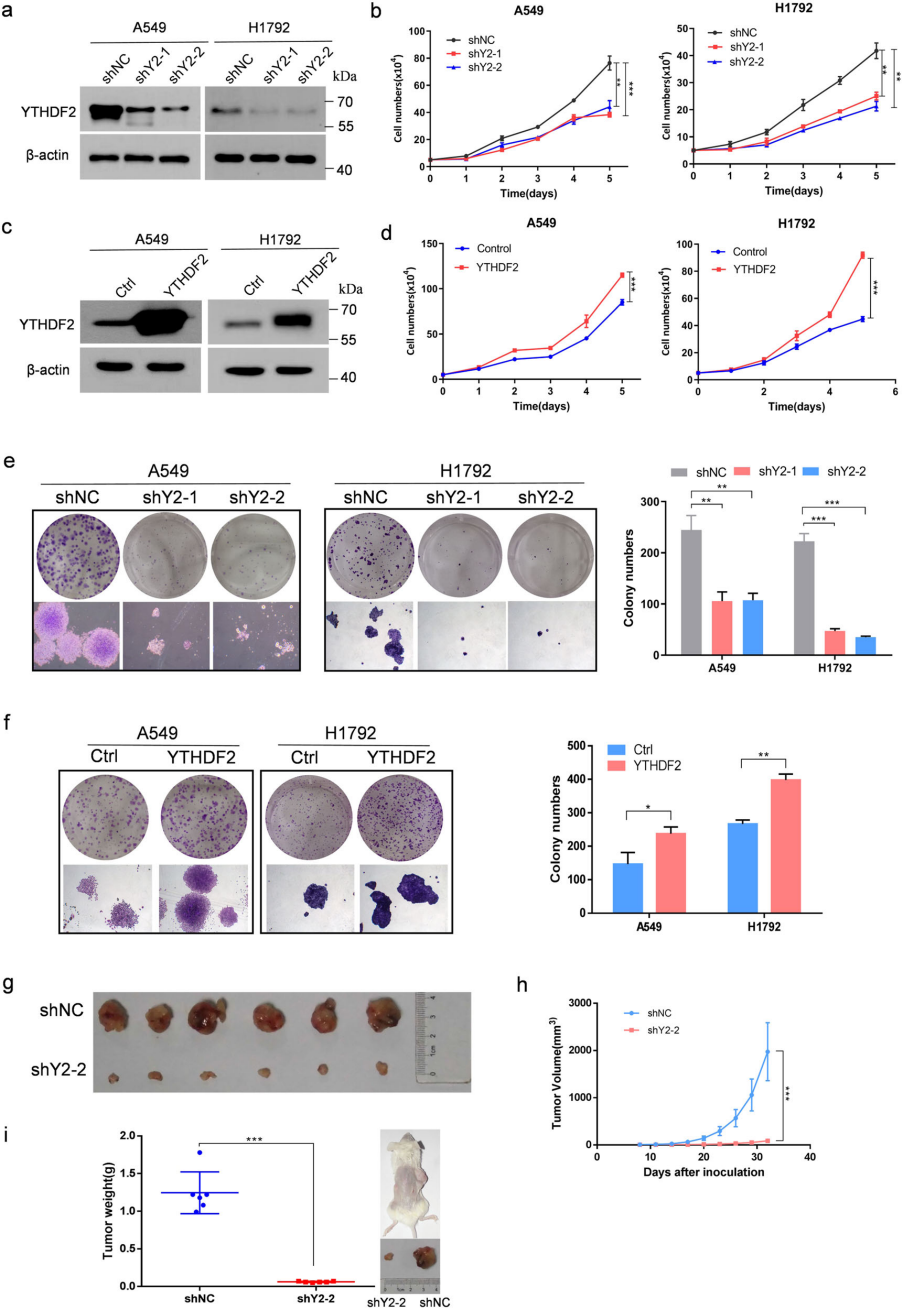
YTHDF2 regulated LUAD cell proliferation and viability in vitro and vivo. **a** Western blot analysis of YTHDF2 expression in A549 and H1792 cells infected with two independent shRNAs targeting YTHDF2 or a control shRNA. **b** Trypan blue live cell count assays were performed to determine cell growth after YTHDF2 knockdown in A549 and H1792 cells. **c** Western blot analysis for YTHDF2 expression in A549 and H1792 cells that were lentivirally infected with YTHDF2 or a control vector. **d** Trypan blue live cell count assays were performed to determine cell growth after YTHDF2 was over-expressed in A549 and H1792 cells. **e** Colony formation assays of A549 and H1792 cells described in **a**. **f** Colony formation assays of A549 and H1792 cells described in **c**. A549 cells with empty vector or YTHDF2 knockdown vectors were subcutaneously injected into nude mice. **h** Tumor size was measured every 3 days, and growth curves were plotted. Tumors were dissected from nude mice of each group and photographed at 32 days after transplantation, and the size and weight of tumors were measured (**g**, **i**). All data and error bars are presented as the mean ± SDs. *P < 0.05, **P < 0.01, and ***P < 0.001 compared with control cells.

Gain-of-function and loss-of-function studies were performed to investigate the pathological role of YTHDF2 in LUAD. As shown in Fig. 2a, the endogenous expression of YTHDF2 was efficiently knocked down by two independent shRNAs (shYTHDF2-1 and shYTHDF2-2) in A549 and H1792 LUAD cells. The knockdown of YTHDF2 significantly suppressed the growth/proliferation of A549 and H1792 cells as determined via trypan blue live cell count assays (Fig. 2b). However, YTHDF2 overexpression via lentiviral transduction significantly enhanced the growth/proliferation of A549 and H1792 cells (Fig. 2c, d). Similarly, colony formation assays revealed that clonogenic capacity was remarkably inhibited by the knockdown of YTHDF2 (Fig. 2e); whereas, the overexpression of YTHDF2 exerted opposite effect on A549 and H1792 cells (Fig. 2f).

A human subcutaneous tumor xenograft model was established to evaluate the role of YTHDF2 attenuation in LUAD tumorigenicity in vivo. First, YTHDF2-deficient shYTHDF2-A549 lung cancer cells and control cells were subcutaneously injected into the bilateral axilla of six nude mice per group. After 32 days, the mice were sacrificed and isolated the tumors. YTHDF2-knocdown A549 cells grew substantially slower than mock A549 cells, and the average tumor volume and weight of YTHDF2-knocdown A549 cells were markedly smaller compared with mock A549 cells (Fig. 2g–i). Collectively, these results demonstrated that YTHDF2 was essential for LUAD cell growth.

### YTHDF2 regulated the metastatic capacity of lung adenocarcinoma cells

**Fig. 3:**
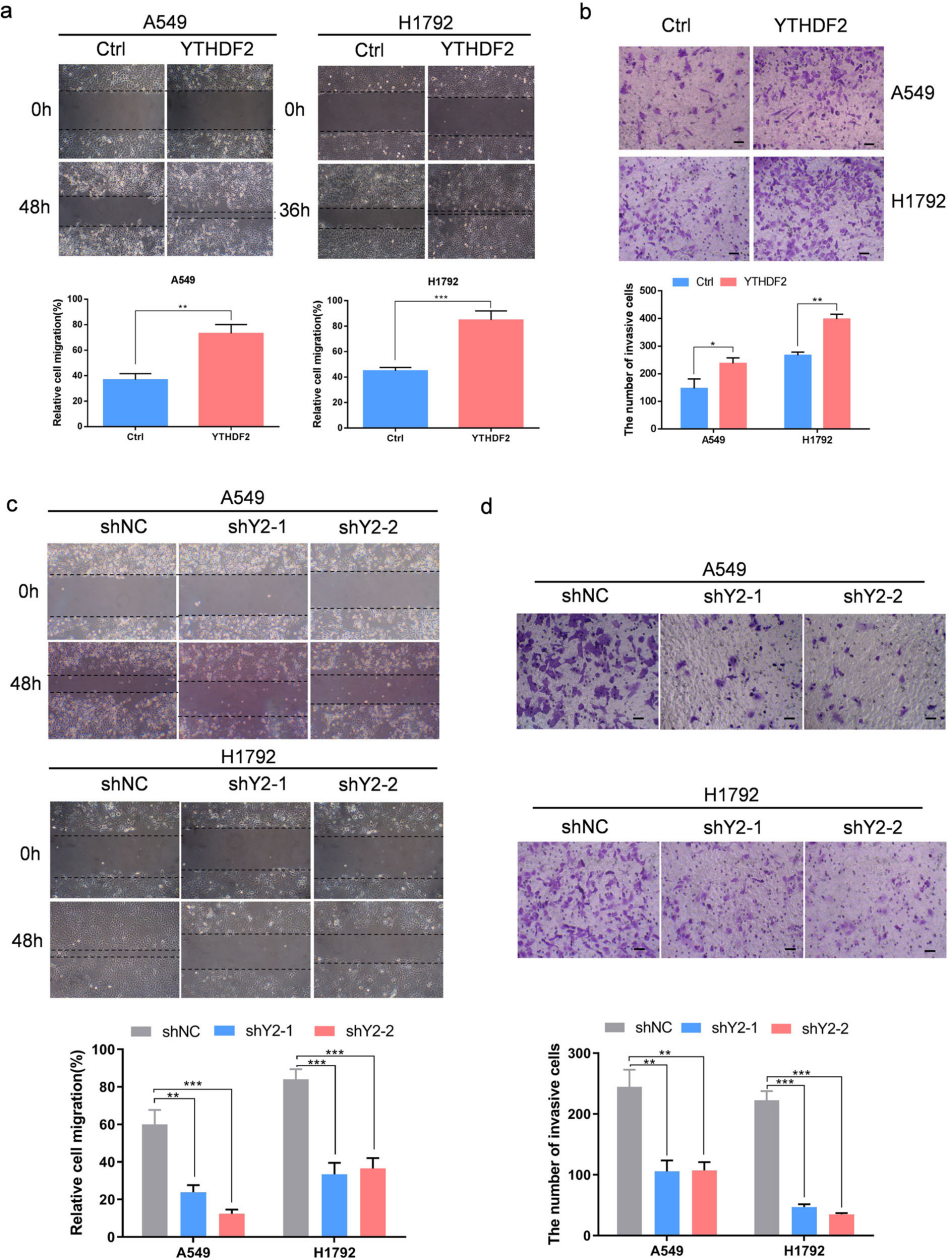
YTHDF2 regulated the metastatic capacity of LUAD cells. **a**, **b** Ectopic overexpression of YTHDF2 significantly promoted A549 and H1792 cell migration and invasive capabilities as examined by wound healing (**a**) and Transwell invasion (**b**) assays. Data are shown as means ± S.D.; *P < 0.05, **P < 0.01, and ***P < 0.001 compared with control cells. **c**, **d** Silencing of YTHDF2 by shRNA effectively inhibited A549 and H1792 cell migration and invasive capabilities. Data are shown as means ± S.D.; **P < 0.01 and ***P < 0.001 compared with control cells.

Next, we investigated the effect of YTHDF2 on the aggressive capacities of LUAD in vitro. Our results showed that the ectopic overexpression of YTHDF2 in A549 and H1792 cells markedly enhanced cell migration capabilities as evidenced by wound healing and Transwell assays (Fig. 3a, b). By contrast, the knockdown of YTHDF2 in A549 and H1792 cells substantially reduced cell migratory speed and invasive capacities (Fig. 3c, d). These results indicated a critical role of YTHDF2 in promoting LUAD metastasis.

To confirm that the observed defects resulted from loss of YTHDF2, we performed rescue experiments. Re-expression of YTHDF2 in YTHDF2-deficient A549 and H1792 cells substantially enhanced the growth/proliferation, restored clonogenic capacity, and migration capabilities of A549 and H1792 cells (Fig. S2a–c).

### Search of potential targets of YTHDF2 through transcriptome-wide m6A-Seq and RNA-Seq assays

**Fig. 4:**
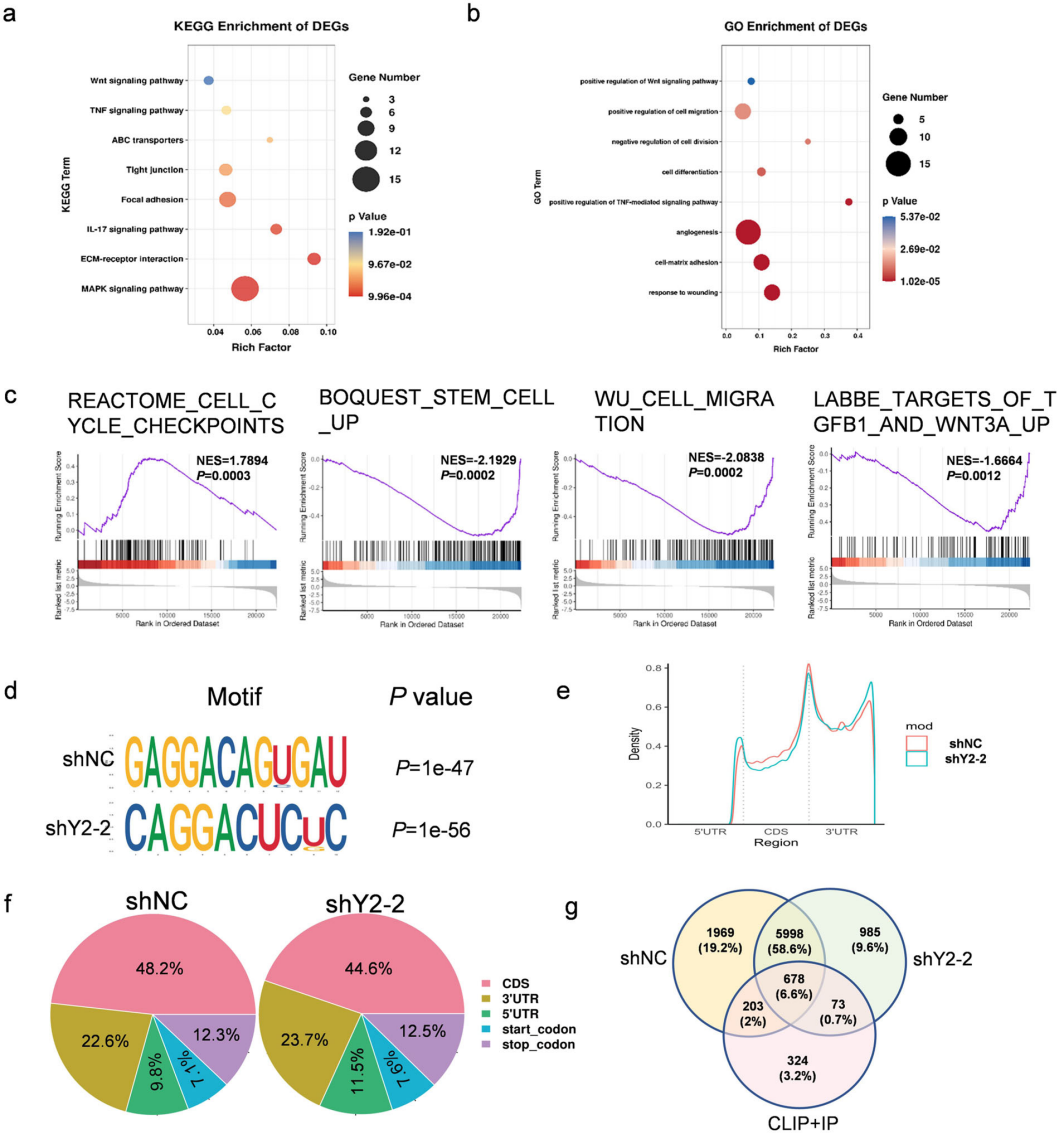
Identification of YTHDF2 targets via MeRIP-seq and RNA-seq in LUAD. **a**, **b** KEGG and GO enrichment analysis of differentially expressed genes (DEGs) identified by RNA-seq. **c** GSEA plots showing that the pathways of DEGs altered by YTHDF2 knockdown were involved in LUAD. **d** m6A motif detected by the HOMER motif analysis with m6A-seq data in A549 cells with or without YTHDF2 knockdown. **e** Metagene profiles of m6A enrichment across the mRNA transcriptome in LUAD. **f** Graphs of m6A peak distribution showing the proportion of total m6A peaks in the indicated regions in control and YTHDF2-knockdown cells. **g** Venn diagram illustrating the overlapped targets of YTHDF2 in control and YTHDF2-knockdown cells identified by m6A-seq analysis and the targets identified by eCLIP and RIP.

Transcriptome-wide m6A-Seq and RNA-Seq assays were then performed to further understand the mechanism by which YTHDF2 influenced LUAD function. The knockdown of YTHDF2 resulted in deregulated gene expression with 126 upregulated and 291 downregulated genes in A549 cells (Supplementary Table S3). KEGG analysis indicated that the significantly altered pathways were MAPK signaling pathway and Wnt signaling pathway, and genes associated with focal adhesion and ABC transporters. Gene Ontology analysis revealed that differentially expressed genes were related to cell matrix adhesion, angiogenesis, cell adhesion, cell differentiation, proliferation, and migration. Moreover, gene set enrichment analysis (GSEA) revealed that the genes altered by YTHDF2 knockdown were associated with cell cycle, stem cell, cell migration, and the Wnt signaling pathway (Fig. 4a–c and Supplementary Fig. s3a), thus providing evidence for critical roles of YTHDF2 in tumorigenesis and the metastasis of LUAD cells.

Next, we mapped the m6A methylomes of shCtrl and shYTHDF2-2 A549 cells by m6A-seq. We identified 11,307 m6A peaks out of 6275 genes in shCtrl A549 cells and 10,994 m6A peaks out of 6038 genes in shYTHDF2-2 A549 cells. These m6A modifications were preferentially located in protein-coding transcripts (48.2% in shNC and 44.6% in shY2-2) and enriched near the stop codons and 3′-UTRs (22.6% in shNC and 23.7% in shY2-2) (Fig. 4d, e). Consistent with other m6A-seq results, we found that m6A peaks were abundant in the mRNA open reading frame, around the stop codons and 3′-UTRs (Fig. 4f). We refered to the PAR-CLIP and RIP-seq results reported by Wang et al. to further explore YTHDF2 targets in LUAD^28^. Interestingly, the data from RNA-seq, m6A-seq, PAR-CLIP, and RIP-seq revealed 678 overlapping genes bound by YTHDF2 which were tagged with m6A. In addition, functional annotation showed that these 678 genes were generally involved in the Wnt signaling pathway (Fig. s3b, c and Table S4).

Given that the Wnt/β-catenin signaling pathway is related to cancer cell proliferation and migration, we hypothesized that the deprivation of the m6A methylation reader YTHDF2 might inhibit tumor growth and migration via the inactivation of the Wnt/β-catenin signaling pathway. Hence, the Wnt/β-catenin signaling pathway was selected for further research.

### AXIN1 mRNA was the target of YTHDF2

**Fig. 5:**
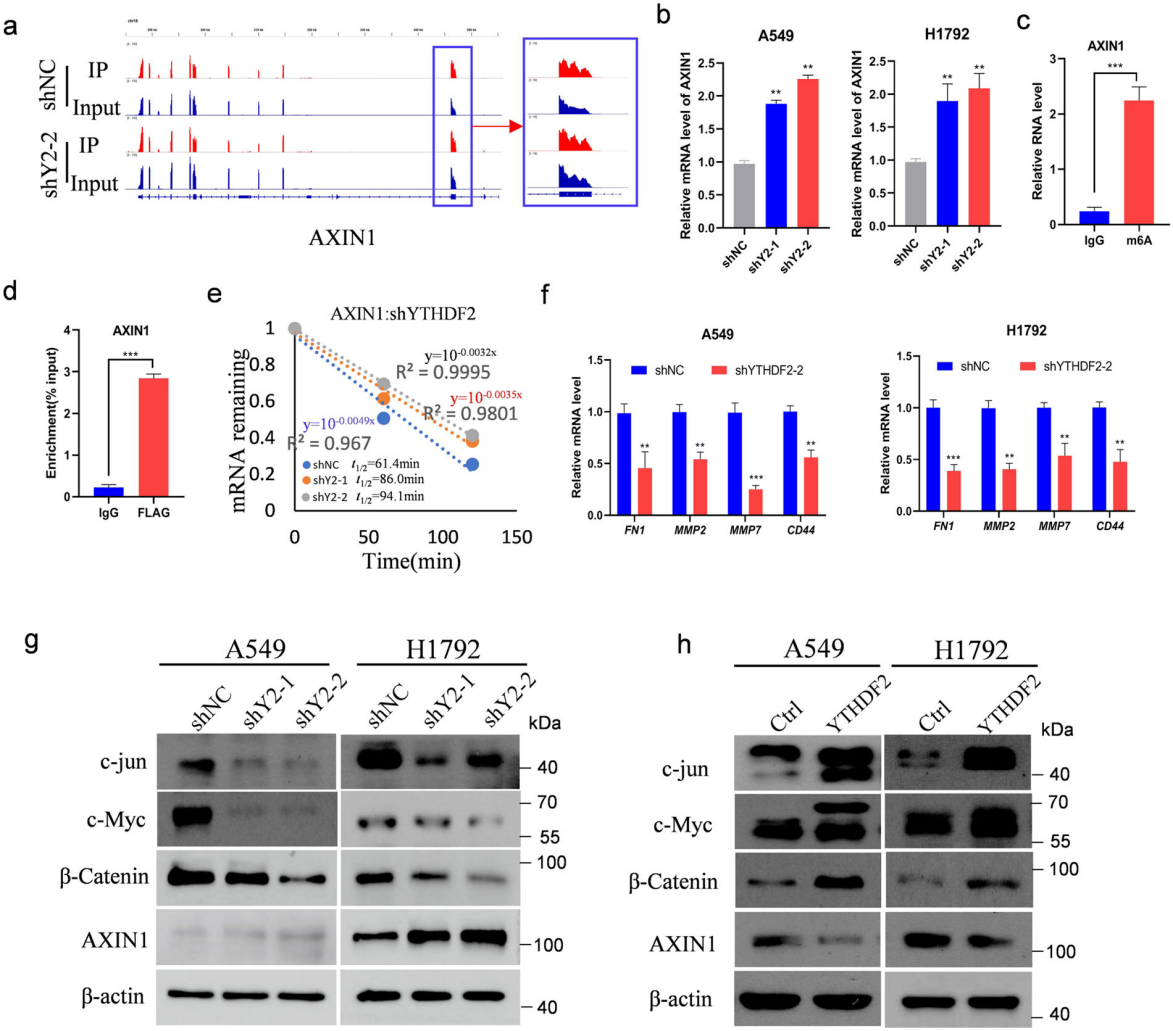
AXIN1/Wnt/β-catenin signaling was targeted by YTHDF2. **a** Distribution of m6A peaks across *AXIN1* transcripts in control and YTHDF2-deficient cells. **b** qPCR analysis of *AXIN1* mRNA expression in A549 and H1792 with or without YTHDF2 knockdown. Samples were normalized to β-actin mRNA. **c** Enrichment of m6A modification in AXIN1 as detected by a gene-specific m6A qPCR assay. **d** RIP–qPCR showing the association of AXIN1 with FLAG-tagged YTHDF2 in A549 cells. **e** Increased AXIN1 mRNA half-life by silencing YTHDF2 in A549 cells. Values were the mean ± S.D. of *n* = 3 independent experiments. **f** Relative mRNA levels of AXIN1/Wnt/β-catenin downstream targets identified by qPCR analysis in A549 and H1792 cells. Data represented means ± S.D. for three independent experiments. Immunoblotting to measure c-jun, c-Myc, β-catenin, and AXIN1 protein levels in transformed A549 and H1792 control cells and cells with YTHDF2 knockdown (**g**) or YTHDF2 overexpression (**h**).

Given that YTHDF2 promotes mRNA degradation by targeting m6A-modified mRNAs to processing bodies in the cytoplasm, identifying the key factor that inactivates the Wnt/β-catenin signaling pathway was important. Among the 678 tagged genes, AXIN1, which encodes a cytoplasmic protein that functions as a negative regulator of Wnt/β-catenin signaling pathway, was selected for systematic study. The m6A levels of AXIN1 were significantly increased in YTHDF2-knockdown cells (Fig. 5a). Then, the transcription and translation of AXIN1 upon YTHDF2 knockdown were assessed. As expected, Knockdown of YTHDF2 increased the mRNA and protein levels of AXIN1 in A549 and H1792 lung cancer cells; whereas, the overexpression of YTHDF2 decreased the protein AXIN1 in A549 and H1792 cells (Fig. 5b, g, h). We further examined the m6A modification status of AXIN1 mRNA through the gene-specific m6A assay, and found a significant enrichment of m6A in AXIN1 mRNA (Fig. 5c). Besides, RIP-qPCR confirmed the interaction between YTHDF2 and AXIN1 mRNA in A549 cells (Fig. 5d). Provided that YTHDF2 targets tend to have short half-lives, we performed mRNA stability profiling on YTHDF2-knockdown and control A549 cells. The median half-life of AXIN1 was significantly extended in YTHDF2-silenced cells relative to that in control cells (Fig. 5e).

We then detected the mRNA expression of FN1, MMP2, MMP7, and CD44, direct target genes of the Wnt/β-catenin signaling pathway, to verify whether AXIN1/Wnt/β-catenin pathway was involved in YTHDF2-silencing mediated progression of LUAD. The knockdown of YTHDF2 decreased the expression of these genes in A549 and H1792 cells (Fig. 5f). Meanwhile, we tested the protein expression of c-jun, c-Myc, and β-catenin in the presence and absence of YTHDF2 knockdown through western blot analysis. The results showed that YTHDF2 knockdown decreased the expression of these genes in A549 and H1792 cells; whereas, YTHDF2 overexpression increased the expression of these proteins in A549 and H1792 cells (Fig. 5g, h). Simultaneously, overexpression of YTHDF2 recovered the protein levels of β-catenin, c-Jun, and c-Myc to varying degrees which were decreased by YTHDF2 silencing (Fig. S2d). Taken together, these results demonstrated that the knockdown of YTHDF2 inhibited Wnt/β-catenin signaling and its downstream targets by upregulating AXIN1mRNA, thereby preventing the progression of malignant lung cancer cells.

### Knockdown of AXIN1 rescues the tumor suppressive effect of YTHDF2 silencing in LUAD cells

**Fig. 6:**
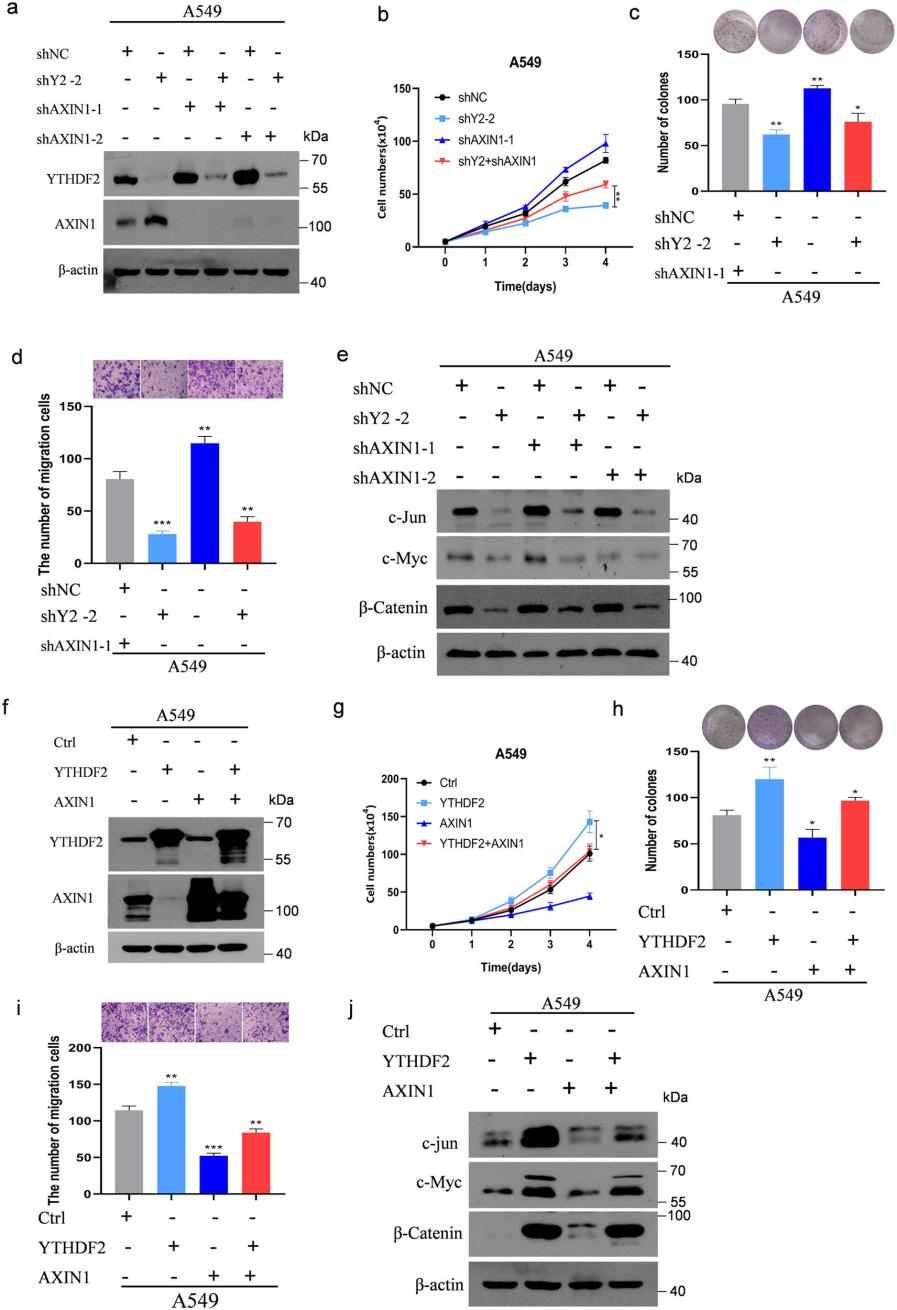
AXIN1 was critical to YTHDF2-promoted Wnt/β-catenin signaling. **a** Immunoblotting of lysates from A549 cells transfected with shNC, shYTHDF2#2, shYTHDF2#2, and/or shAXIN1-1 or shAXIN1-2. Expression levels of YTHDF2 and AXIN1 were measured. β-actin was used as a loading control. **b** Live cell count, **c** colony formation, **d** migration, and **e** immunoblotting of AXIN1/Wnt/β-catenin downstream targets in cells described in **a**. **f** Immunoblotting of lysates from A549 cells transfected with shCtrl, YTHDF2, AXIN1, YTHDF2, and/AXIN1 vector. Expression levels of YTHDF2 and AXIN1 were measured. β-actin was used as a loading control. **g** Live cell count, **h** colony formation, **i** migration, and **j** immunoblotting of AXIN1/Wnt/β-catenin downstream targets in cells described in **f**.

**Fig. 7:**
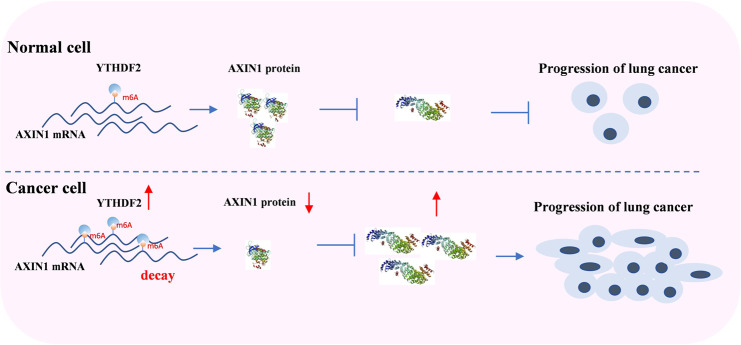
YTHDF2 high expressed promoted progression and metastasis of lung cancer cells via AXIN1/Wnt/β-catenin signalling. YTHDF2 facilitated the decay of AXIN1 m6A mRNA and results in the dereased AXIN1protein level.The decreased AXIN1 promote the activity of Wnt/β-catenin signalling and enhance the proliferation and migration of lung cancer.

We first determined whether AXIN1 knockdown could reverse the effects of YTHDF2 silencing to ascertain, whether AXIN1 was a vital contributor to the function of YTHDF2 in LUAD tumorigenesis and metastasis. Stable A549 cells with YTHDF2-silengcing vectors and/or AXIN1 knockdown were thus established (Fig. 6a). As anticipated, the knockdown of AXIN1 expression abrogated the inhibition of proliferation caused by the knockdown of YTHDF2. Additionally, the colony formation assay provided similar results. Specifically, this assay showed that AXIN1 knockdown almost completely recovered the colonogenic capabilities of A549 cells that were hampered by the knockdown of YTHDF2 (Fig. 6b, c). Likewise, the migration assay showed that AXIN1 deficiency largely abrogated the inhibitory effects of YTHDF2 knockdown on these malignant behaviors in LUAD (Fig. 6d). Furthermore, AXIN1 knockdown recovered the protein levels of β-catenin decreased by YTHDF2 silencing, and c-Myc and c-Jun were also recovered to varying degrees (Fig. 6e). Conversely, the forced expression of AXIN1 partially abolished the oncogenic phenotypes and Wnt/β-catenin signaling activation induced by YTHDF2 overexpression in A549 cells. These results indicated that the cell proliferation, clone formation, and metastasis caused by the overexpression of YTHDF2 were inhibited, and the increased expression levels of β-catenin, c-Jun, and c-Myc proteins caused by the overexpression of YTHDF2 also decreased (Fig. 6f–j). Taken together, these results suggested that YTHDF2 knockdown inhibited LUAD cancer tumorigenesis and metastasis by increasing AXIN1 expression and suppressing Wnt/β-catenin signaling (Fig. 7).

## Discussion

The disorder of m6A modification on RNA is closely related to the occurrence and development of various cancers. Some studies also reported that the m6A methyltransferases (writers), demethylases (erasers), and readers can either enhance or suppress tumorigenesis in different cancers^29^. Although a previous study from a metabolic perspective showed that YTHDF2 deficiency suppresses lung cancer growth through 6PGD inhibition^20^, the molecular mechanisms of YTHDF2 in lung adenocarcinoma proliferation and metastasis remain largely unclear. In this study, we found that the YTHDF2 gene was frequently upregulated in LUAD in TCGA, CHOICE, and GEO cohorts. In addition, the expression of YTHDF2 is also increased in many different cancers, suggesting that YTHDF2 might be an important oncogene. Notably, analyzing TCGA survival data revealed that high YTHDF2 expression had no association with overall survival, indicating that the mRNA expression of YTHDF2 was not a prognostic marker for overall survival in patients with LUAD. However, further studies are needed to determine whether changes in YTHDF2 protein levels are related to prognosis. In this study, we demonstrated that YTHDF2 was vital for the proliferation and metastasis of the LUAD cell lines A549 and H1792 in vitro and/or in vivo. Taken together, our data indicated that YTHDF2 acts as a tumor promoter in LUAD.

We adopted a multiomics screening strategy by combining m6A-seq, RNA-seq, and the published data of RIP-seq and CLIP-seq to dissect the mechanisms of YTHDF2 in LUAD. We first analyzed the m6A methyl body in LUAD cells and screened the potential targets of YTHDF2. Consistent with published results, our m6A-seq results showed that YTHDF2-binding sites were distributed in coding and untranslated regions, and YTHDF2 preferentially bonded to the sites that were enriched near the stop codon and 3′ UTR. In order to find specific targets effectively, we overlapped the RNA-seq and m6A-seq results with previously published CLIP and RIP-seq genes, and the result showed that 678 genes bound by YTHDF2 were marked with m6A, and the functional annotation showed these potential direct target genes participated in the Wnt signaling pathway.

The Wnt/β-catenin pathway is a highly conserved pathway, and its abnormal activation promotes the progression of a variety of cancers by faciliated cell growth and metastasis. In addition, it also promotes the occurrence of tumor cell stemness^30,31^. More and more evidences support the epigenetic regulation of Wnt/β-catenin signaling in cancer, although the effect of mRNA modification remains inadequately studied. The relationship between RNA modification and tumor-related pathways, including the Wnt signaling pathway, has only recently been fully understood with the development of meRIP sequencing. In hepatoblastoma, m6A methylation usually activates the Wnt/catenin pathway by promoting the expression of CTNNB1^32^. METTL3 modulates the target gene m6A (such as LRP6 and dishevell 1) to regulate Wnt signaling, thereby changing the viability, proliferation, migration, and tube formation of endothelial cells^33^. YTHDF1 promotes the translation of a key Wnt receptor frizzled7 (FZD7) in an m6A-dependent manner, and mutated YTHDF1 enhances the expression of FZD7, leading to the hyperactivation of the Wnt/β-catenin pathway and the promotion of gastric carcinogenesis^34^. YTHDF1 plays an indispensable role in the activation of Wnt/β-catenin to sustain intestinal stem cell stemness characteristics during regeneration and tumorigenesis^35^. METTL3 promotes osteosarcoma cell progression by upregulating the m6A level of LEF1 and activating the Wnt/β-catenin signaling pathway^36^. ALKBH5 attenuates PDAC cell proliferation and metastasis by downregulating the m6A level of WIF-1 to inhibit Wnt signaling^37^. In addition, previous studies have shown that in the cytoplasm, YTHDF1 promotes the translation of its target by recruiting initiating factors and promoting ribosome loading^8^. However, YTHDF2 easily induces the degradation of its targets by locating these targets to the processed bodies^28^. Therefore, we speculated that YTHDF2 activates the wnt pathway to promote cell proliferation and metastasis, possibly by targeting inhibitors of the WNT pathway and inducing its degradation. In our research, by using multistep screening and validation methods, we further confirmed that AXIN1 is a vital target of YTHDF2 and that its regulation is m6A dependent. AXIN1 directly interacts with all other core components of the destruction complex (β-catenin, APC, CKa, and GSK3) and is thus the central scaffold of the complex and the rate-limiting factor of the β-catenin destruction complex. The degradation of *AXIN1* in Wnt-activated cells is considered to be the immediate cause of β-catenin stabilization^38^. Therefore, our study provided a different perspective of the target AXIN1, which controls its translation by manipulating YTHDF2 or cellular m6A levels. In addition, our research demonstrated that the overexpression of AXIN1 could rescue malignant phenotypes caused by the upregulation of YTHDF2 in LUAD and vice versa. Indeed, we have discovered many other candidate targets of YTHDF2, which are also involved in cancer growth and metastasis. YTHDF2 might regulate the expression of multiple genes, and the effect of YTHDF2 on LUAD could have a global effect on multiple targets. However, this effect needs further verification.

In summary, we showed that YTHDF2 deficiency inhibited lung cancer cell proliferation and migration by modulating m6A levels of AXIN1, and impeding the activation of Wnt/β-catenin signaling. These findings illustrated the underlying mechanism of LUAD tumorigenesis and migration regulated by m6A-modified reader, and provided a new direction for the development of effective therapeutic strategies for the treatment of lung adenocarcinoma.

## Materials and methods

### Reagents and antibodies

Antibodies against YTHDF2 (catalog number: 24744-I-AP) was purchased from Proteintech (Wuhan, Hubei, P.R.C). Antibodies against AXIN1, β-catenin, β-actin, c-jun, c-Myc, and secondary antibodies were purchased from Cell Signaling Technology (Danvers, MA, USA). Protein G Sepharose 4 Fast Flow was acquired from GE.

### Cell culture and tissue samples

The human LUAD cell lines A549 and H1792 were cultured in RPMI 1640 medium supplemented with 10% fetal bovine serum (FBS, Gibco, Thermo Scientific, Grand Island, NY, USA). Human embryonic kidney-derived cells HEK293T were cultured with Dulbecco’s modified Eagle medium (DMEM) with 10% FBS. Cells were cultured at 37 °C in a humidified incubator containing 5% CO_2_. All cells were purchased from the Cell Bank of the Chinese Academy of Sciences (Shanghai, China). All cells were commercially authenticated by short tandem repeats genotyping (Guangzhou Jenniobio Co., Ltd, Guangzhou, China), and tested for mycoplasma contamination in March 2020. Primary LUAD tumors and adjacent normal tissues of eight patients were used. The study was approved by the First Affiliated Hospital of Jinan University Ethics Review Board in accordance with the Declaration of Helsinki. Informed consent was obtained from each participant.

### Generation of stable knockdown and overexpression cell lines

Endogenous YTHDF2 was stably knocked down using lentiviral vectors containing shRNA constructs (shYTHDF2#1, 5′-TACTGATTAAGTCAGGATTAA-3′, shYTHDF2#2, 5′-ATGGATTAAACGATGATGAT-3′). H1792 and A549 cells with AXIN1 depleted by (shAXIN1#1, 5′-CACCGGTTCAGGTGAGTCCGACGCC-3′, shAXIN1#2, 5′- CACCGGCCTTCGCTGTACCGTCTAC-3′). Ectopic YTHDF2 was stably overexpressed by using pLVX-puromycin. Lentivirus was produced according to the manufacturer’s instructions. In brief, HEK293T cells were seeded the day before transfection. When the cells reaches 70–90% confluent, the target plasmid and the packaging plasmid psPAX2 and envelope plasmid pMD2.G were co-transfected into HEK293T cells through Lipofectamine™ 3000 Transfection reagent (Life Technologies, Carlsbad, USA). After 48–72 h, the lentivirus-containing supernatant medium was collected, filtered, concentrated, and used to infect target cells. After 48 h, puromycin was added to the target cells for resistance screening. The knockdown of endogenous proteins and overexpression of ectopic proteins were confirmed through western blot analyses.

### Cell growth assay

The LUAD cells were cultured in six-well plates (5 × 10^4^ cells in each well) for proliferation assay, and at least three multiple wells in each group. Cell growth was measured based on the cell numbers recorded for five consecutive days after seeding.

### Wound healing assay in vitro

5 × 10^5^ LUAD cells were seeded on a six-well plate the day before the experiment. The cells were scratched with a 20 µl pipette tips when the cells were almost 100% confluent. To reduce the effect of cell proliferation, serum-free medium was used. Wound areas were photographed at 0 h and 36 or 48 h by using a microscope. Then the wound area could be calculated by using Image J to manually track the cell-free area of the captured image. Migration rates were quantified by the percentage of area reduction.

### Xenograft lung cancer mouse model

Four-to-five-week-old NOD/SCID male mice were purchased from Beijing Charles River Laboratories. A total of 1 × 10^6^ A549 shYTHDF2 or vector cells diluted in 0.1 ml of PBS were injected into the axilla of six NOD/SCID mice per group. After growth for another 32 days, the mice were sacrificed and isolated the tumors. All experimental protocols were in accordance with the Jinan University Institutional Animal Care and Use Committee.

### Quantitative real-time polymerase chain reaction assay

Total RNA was extracted from cells by using Trizol Reagent (Takara, Dalian, China) in accordance with the manufacturer’s protocol. The PrimeScriptTM RT reagent kit with gDNA eraser (Takara, Dalian, China) was used to reverse transcription of RNA to cDNA. Then the reaction system was configured according to protocol of the SYBR Premix Ex TaqTM II kit, and the levels of RNA transcripts were performed by using the Bio-rad CFX96 real-time PCR system (Biorad, USA). The human housekeeping gene β-actin was used as the reference gene control. The sequences of the primers were as follow: Axin1 forward primer, 5′-TGGAGCCCTGTGACTCGAA-3′, Reverse primer, 5′-GGGACACGATGCCATTGTTATC-3′; FN1 forward primer, 5′-CGGTGGCTGTCAGTCAAAG-3′, Reverse primer, 5′-AAACCTCGGCTTCCTCCATAA-3′; MMP2 forward primer, 5′-GATACCCCTTTGACGGTAAGGA-3′, Reverse primer, 5′-CCTTCTCCCAAGGTCCATAGC-3′; MMP7 forward primer, 5′-GAGTGAGCTACAGTGGGAACA-3′, Reverse primer, 5′-CTATGACGCGGGAGTTTAACAT-3′; CD44 forward primer, 5′-CTGCCGCTTTGCAGGTGTA-3′, Reverse primer, 5′-CATTGTGGGCAAGGTGCTATT-3′. The qRT-PCR conditions were as follows: one cycle at 95 °C for 30 s, 40 cycles at 95 °C for 5 s, 60 °C for 30 s, and then dissociation stage.

### Western blot analysis

The cells were lysed with RIPA on ice for 10 min and then centrifuged at 10,000 rpm at 4 °C for 15 min. The concentrations of protein samples were detected by BCA assay (P0012, Beyotime, China). The protein samples were separated through SDS-polyacrylamide gel electrophoresis and then transferred onto PVDF membranes (Millipore). The membranes were blocked with 5% skimmed milk for 1 h and then incubated on the shaker overnight at 4 °C with the primary antibody, and the secondary antibody was incubated at room temperature for about 1 h. Signals were detected by using an ECL Western blotting detection kit (Thermo Fisher Scientific).

### Methylated RNA immunoprecipitation sequencing (MeRIP-seq)

Total RNA was isolated and purified by using TRIzol reagent (Invitrogen, Carlsbad, CA, USA) in accordance with the manufacturer’s procedure. Poly (A) RNA was purified from 50 μg total RNA using Dynabeads Oligo (dT) and then was fragmented into small pieces using Magnesium RNA Fragmentation Module (NEB, cat.e6150, USA) under 86 °C for 7 min. Then the cleaved RNA fragments were incubated for 2 h at 4 °C with m6A-specific antibody (No. 202003, Synaptic Systems, Germany) in IP buffer. Eluted m6A-containing fragments (IP) and untreated input control fragments were converted to final cDNA library following a strand-specific library preparation by dUTP method. The average size for the paired-end libraries was ~300 ± 50 bp. At last, we performed the 2 × 150 bp paired-end sequencing (PE150) on an illumina Novaseq™ 6000 (LC-Bio Technology CO., Ltd., Hangzhou, China) following the vendor’s recommended protocol.

### Bioinformatics analysis of m6A-seq and RNA-seq data

We used HISAT2 (http://daehwankimlab.github.io/hisat2) to map reads to the genome of *Homo sapiens* (Version: GRCh38) with default parameters. Mapped reads of IP and input libraries were provided for R package exomePeak, which identifies m6A peaks with bed or bam format that could be adapted for visualization on the UCSC genome browser or IGV software (http://www.igv.org/). HOMER were used for de novo and known motif finding, followed by localization of the motif with respect to peak summit. Called peaks were annotated by intersection with gene architecture using R package ChIPseeker. Then StringTie was used to perform expression level for all mRNAs from input libraries by calculating FPKM (FPKM = [total_exon_fragments/ mapped_reads(millions) × exon_length (kB)]). The differentially expressed mRNAs were selected with log2 (fold change) >1 or log2 (fold change) <−1 and *P* value < 0.05 by R package edgeR.

### RNA immunoprecipitation reverse-transcription quantitative polymerase chain reaction assays

RNA immunoprecipitation was implemented as previously described with some modifications. Briefly, 1 μg equivalent of m6A or FLAG antibody was conjugated to protein G-conjugated agarose beads (Millipore) on a shaker overnight at 4 °C. A 100 μg equivalent of total RNA was incubated with the antibody in immunoprecipitation buffer (150 mM NaCl, 50 mM Tris-HCl pH 7.5, and 0.5% Triton100) supplemented with RNase inhibitor for 2–3 h at 4 °C. After centrifugation and discarding the supernatant, the sediment was incubated with 300 µl of elution buffer (5 mM Tris-HCl pH 7.5, 1 mM ethylenediaminetetraacetic acid, and 0.05% sodium dodecyl sulfate) supplemented with 2 µl of proteinase k at 50 °C for 1.5 h to elute RNA from the beads. Input and co-immunoprecipitated RNAs were recovered through TRIzol extraction and analyzed via qRT-PCR.

### RNA stability assay and sequencing for mRNA lifetime

After 24 h, A549 and H1792 cells stably expressing shRNA against YTHDF2 or shNS were seeded into six-well plates to 50% confluence. Cells were treated with 5 μg/ml actinomycin D and collected at the indicated time points. Total RNA was extracted by using TRIzol (Takara), and AXIN1 mRNA levels were determined through qRT-PCR. The turnover rate and half-life of mRNA were assessed in accordance with anteriorly reported methods^39^.

### Statistical analysis

Statistical analyses were performed by using SPSS 20.0, GraphPad Prism 6.0, or R 3.6.1 software. Student’s *t*-test or two-way analysis of variance, Pearson *χ*^2^ test, Kaplan–Meier curve with log-rank test were conducted as indicated. Figures were done through GraphPad Prism 6.0 or R 3.6.1 software. Data were expressed as the mean ± standard deviation (S.D.) of at least three independent experiments. *P* ≤ 0.05 was regarded as statistically significant.

## Supplementary information

Supplement legends

Figure s1,s2,s3

Table s1,s2

Table S3

Table S4

## Data Availability

The raw data stored in the GEO (GSE161090).

## References

[1] Torre LA (2015). Global cancer statistics, 2012. Cancer J. Clin..

[2] Ettinger DS (2019). NCCN guidelines insights: non-small cell lung cancer, version 1.2020. J. Natl Compr. Cancer Netw..

[3] Miller KD (2019). Cancer treatment and survivorship statistics, 2019. Cancer J. Clin..

[4] Siegel RL, Miller KD, Jemal A (2019). Cancer statistics, 2019. Cancer J. Clin..

[5] Koch A (2018). Analysis of DNA methylation in cancer: location revisited. Nat. Rev. Clin. Oncol..

[6] Kraiczy J (2019). DNA methylation defines regional identity of human intestinal epithelial organoids and undergoes dynamic changes during development. Gut.

[7] Roundtree IA, Evans ME, Pan T, He C (2017). Dynamic RNA modifications in gene expression regulation. Cell.

[8] Wang X (2015). N(6)-methyladenosine modulates messenger RNA translation efficiency. Cell.

[9] Lin S, Choe J, Du P, Triboulet R, Gregory RI (2016). The m(6)A methyltransferase METTL3 promotes translation in human cancer cells. Mol. Cell.

[10] He L (2019). Functions of N6-methyladenosine and its role in cancer. Mol. Cancer.

[11] Huang H, Weng H, Chen J (2020). The biogenesis and precise control of RNA m(6)A methylation. Trends Genet..

[12] Chen M (2018). RNA N6-methyladenosine methyltransferase-like 3 promotes liver cancer progression through YTHDF2-dependent posttranscriptional silencing of SOCS2. Hepatology.

[13] Ma JZ (2017). METTL14 suppresses the metastatic potential of hepatocellular carcinoma by modulating N(6)-methyladenosine-dependent primary MicroRNA processing. Hepatology.

[14] Wang Q (2020). METTL3-mediated m(6)A modification of HDGF mRNA promotes gastric cancer progression and has prognostic significance. Gut.

[15] Yue B (2019). METTL3-mediated N6-methyladenosine modification is critical for epithelial-mesenchymal transition and metastasis of gastric cancer. Mol. Cancer.

[16] Deng R (2019). m(6)A methyltransferase METTL3 suppresses colorectal cancer proliferation and migration through p38/ERK pathways. Onco.Targets Ther..

[17] Zaccara S, Ries RJ, Jaffrey SR (2019). Reading, writing and erasing mRNA methylation. Nat. Rev. Mol. Cell Biol..

[18] Deng X (2018). RNA N(6)-methyladenosine modification in cancers: current status and perspectives. Cell Res..

[19] Shi H, Wei J, He C (2019). Where, when, and how: context-dependent functions of RNA methylation writers, readers, and erasers. Mol. Cell.

[20] Sheng H (2020). YTH domain family 2 promotes lung cancer cell growth by facilitating 6-phosphogluconate dehydrogenase mRNA translation. Carcinogenesis.

[21] Paris J (2019). Targeting the RNA m(6)A reader YTHDF2 selectively compromises cancer stem cells in acute myeloid leukemia. Cell Stem Cell.

[22] Zhong L (2019). YTHDF2 suppresses cell proliferation and growth via destabilizing the EGFR mRNA in hepatocellular carcinoma. Cancer Lett..

[23] Zhang XC (2019). Comprehensive genomic and immunological characterization of Chinese non-small cell lung cancer patients. Nat. Commun..

[24] Okayama H (2012). Identification of genes upregulated in ALK-positive and EGFR/KRAS/ALK-negative lung adenocarcinomas. Cancer Res..

[25] Su LJ (2007). Selection of DDX5 as a novel internal control for Q-RT-PCR from microarray data using a block bootstrap re-sampling scheme. BMC Genom..

[26] Landi MT (2008). Gene expression signature of cigarette smoking and its role in lung adenocarcinoma development and survival. PLoS ONE.

[27] Wei TY (2012). Protein arginine methyltransferase 5 is a potential oncoprotein that upregulates G1 cyclins/cyclin-dependent kinases and the phosphoinositide 3-kinase/AKT signaling cascade. Cancer Sci..

[28] Wang X (2014). N6-methyladenosine-dependent regulation of messenger RNA stability. Nature.

[29] Huang H, Weng H, Chen J (2020). m(6)A modification in coding and non-coding RNAs: roles and therapeutic implications in cancer. Cancer Cell.

[30] Zhan T, Rindtorff N, Boutros M (2017). Wnt signaling in cancer. Oncogene.

[31] Yang S (2017). FOXP3 promotes tumor growth and metastasis by activating Wnt/beta-catenin signaling pathway and EMT in non-small cell lung cancer. Mol. Cancer.

[32] Liu L (2019). m(6)A mRNA methylation regulates CTNNB1 to promote the proliferation of hepatoblastoma. Mol. Cancer.

[33] Yao MD (2020). Role of METTL3-Dependent N(6)-methyladenosine mRNA modification in the promotion of angiogenesis. Mol. Ther..

[34] Pi, J. et al. YTHDF1 promotes gastric carcinogenesis by controlling translation of FZD7. *Cancer Res*. 10.1158/0008-5472.CAN-20-0066 (2020). 10.1158/0008-5472.CAN-20-0066 32788173

[35] Han B (2020). YTHDF1-mediated translation amplifies Wnt-driven intestinal stemness. EMBO Rep..

[36] Miao W, Chen J, Jia L, Ma J, Song D (2019). The m6A methyltransferase METTL3 promotes osteosarcoma progression by regulating the m6A level of LEF1. Biochem. Biophys. Res. Commun..

[37] Tang B (2020). m(6)A demethylase ALKBH5 inhibits pancreatic cancer tumorigenesis by decreasing WIF-1 RNA methylation and mediating Wnt signaling. Mol. Cancer.

[38] Li VS (2012). Wnt signaling through inhibition of beta-catenin degradation in an intact Axin1 complex. Cell.

[39] Huang H (2018). Recognition of RNA N(6)-methyladenosine by IGF2BP proteins enhances mRNA stability and translation. Nat. Cell Biol..

