# Supplementary material for: RNA m6A reader YTHDF2 facilitates lung adenocarcinoma cell proliferation and metastasis by targeting the AXIN1/Wnt/β-catenin signaling
Source: Cell Death Dis. 2021 May 13;12(5):479. doi: 10.1038/s41419-021-03763-z (PMC8116339; doi:10.1038/s41419-021-03763-z)
Supplement: Supplementary file 1 — Supplement legends [file 41419_2021_3763_MOESM1_ESM.docx]

**Supplements**

Figure s1. m6A WER expression in LUAD and the YTHDF2 expression in different tumors. (a) Histogram of the expression of m6A WERs in the TCGA and CHOICE database of LUAD. (b) Relative RNA levels of YTHDF2 in different tumors in TCGA. (c) Kaplan–Meier analysis of LUAD in GEO (GSE31210, GSE37745, and GSE3141) for correlations between YTHDF2 expression and overall survival.

Figure s2.(a)The protein levels of YTHDF2 in A549 and H1792 cells with YTHDF2 knockdown or YTHDF2 overexpression were measured by western blot.(b) Trypan blue live cell count assays were performed to determine cell growth after YTHDF2 was over-expressed or knockdown or the combine in A549 and H1792 cells. (c) Colony formation assays of A549 and H1792 cells described in (b). (d) Ectopic overexpression of YTHDF2 significantly rescued YTHDF2-silenced A549 and H1792 cell invasive capabilities as examined by Transwell invasion assays. Data are shown as means ± S.D.; **P* < 0.05, ***P* < 0.01 and ****P* < 0.001 compared with control cells. (d) Immunoblotting of lysates from A549 cells transfected with shCtrl, YTHDF2, shYTHDF2-2, YTHDF2, and/shYTHDF2-2 vector. Expression levels of c-jun, c-Myc, β-catenin, and AXIN1 were measured. β-actin was used as a loading control.

Figure s3. (a) GSEA plots showing that the pathways of DEGs altered by YTHDF2 were involved in LUAD. (b–c) KEGG and GO enrichment analysis of the common genes identified by m6A-seq analysis and the targets identified by CLIP and RIP.

Table S1. Correlation analysis for clinicopathologic variables in YTHDF2 expression among 513 lung adenocarcinoma patients in TCGA database

Table S2. Correlation analysis for clinicopathologic variables in YTHDF2 expression among 131 lung adenocarcinoma patients in CHOICE study

Table S3. The regulated gene expression of the loss of YTHDF2 in shYTHDF2 relative to in vector A549 cells.

Table S4. KEGG and GO enrichment analysis of overlapping the genes from RNA-seq, m6A-seq, PAR-CLIP, and RIP-seq.
