# Supplementary material for: RNA m6A reader YTHDF2 facilitates lung adenocarcinoma cell proliferation and metastasis by targeting the AXIN1/Wnt/β-catenin signaling
Source: Cell Death Dis. 2021 May 13;12(5):479. doi: 10.1038/s41419-021-03763-z (PMC8116339; doi:10.1038/s41419-021-03763-z)
Supplement: Supplementary file 2 — Figure s1,s2,s3 [file 41419_2021_3763_MOESM2_ESM.pptx]

## Slide 1
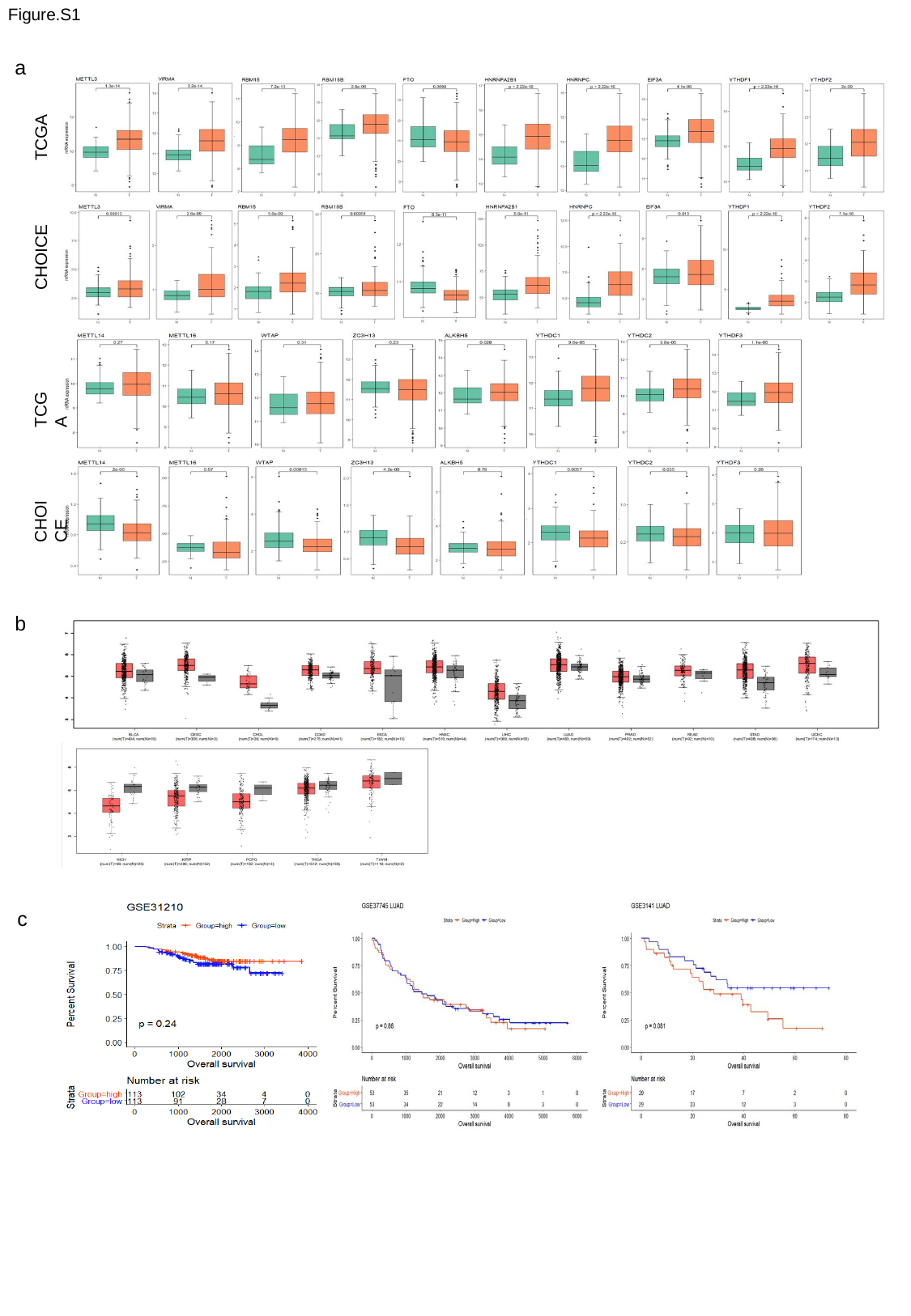

Figure.S1
a
TCGA
CHOICE
TCGA
CHOICE
b
c

## Slide 2
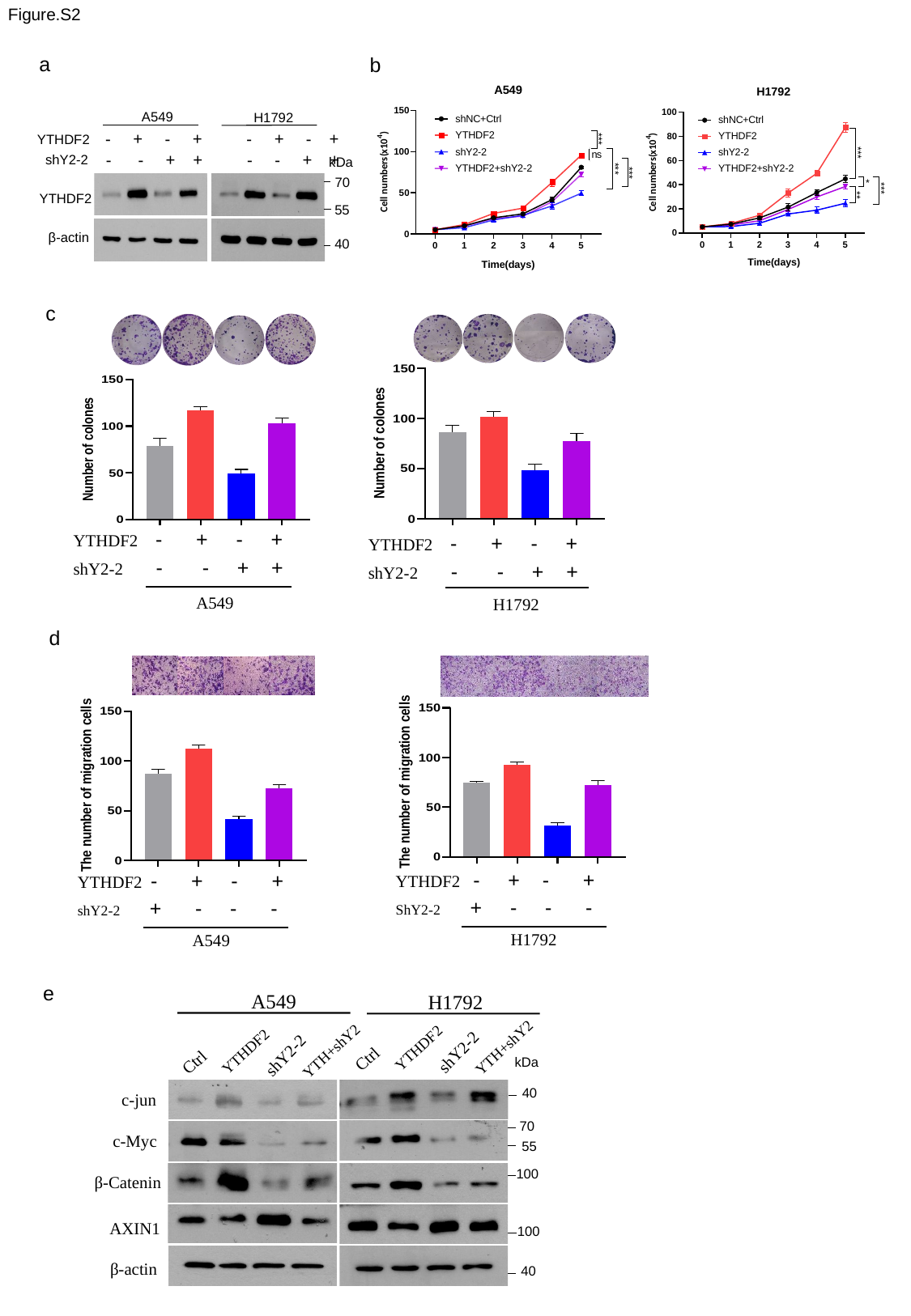

Figure.S2
a
b
A549
H1792
- + - + - + - +
YTHDF2
- - + + - - + +
shY2-2
kDa
70
YTHDF2
55
β-actin
40
c
YTHDF2 - + - +
shY2-2 - - + +
H1792
YTHDF2 - + - +
shY2-2 - - + +
A549
d
YTHDF2 - + - +
ShY2-2 + - - -
H1792
YTHDF2 - + - +
shY2-2 + - - -
A549
e
A549
H1792
YTHDF2
YTHDF2
YTH+shY2
shY2-2
YTH+shY2
shY2-2
Ctrl
Ctrl
kDa
40
c-jun
70
c-Myc
55
100
β-Catenin
AXIN1
100
β-actin
40

## Slide 3
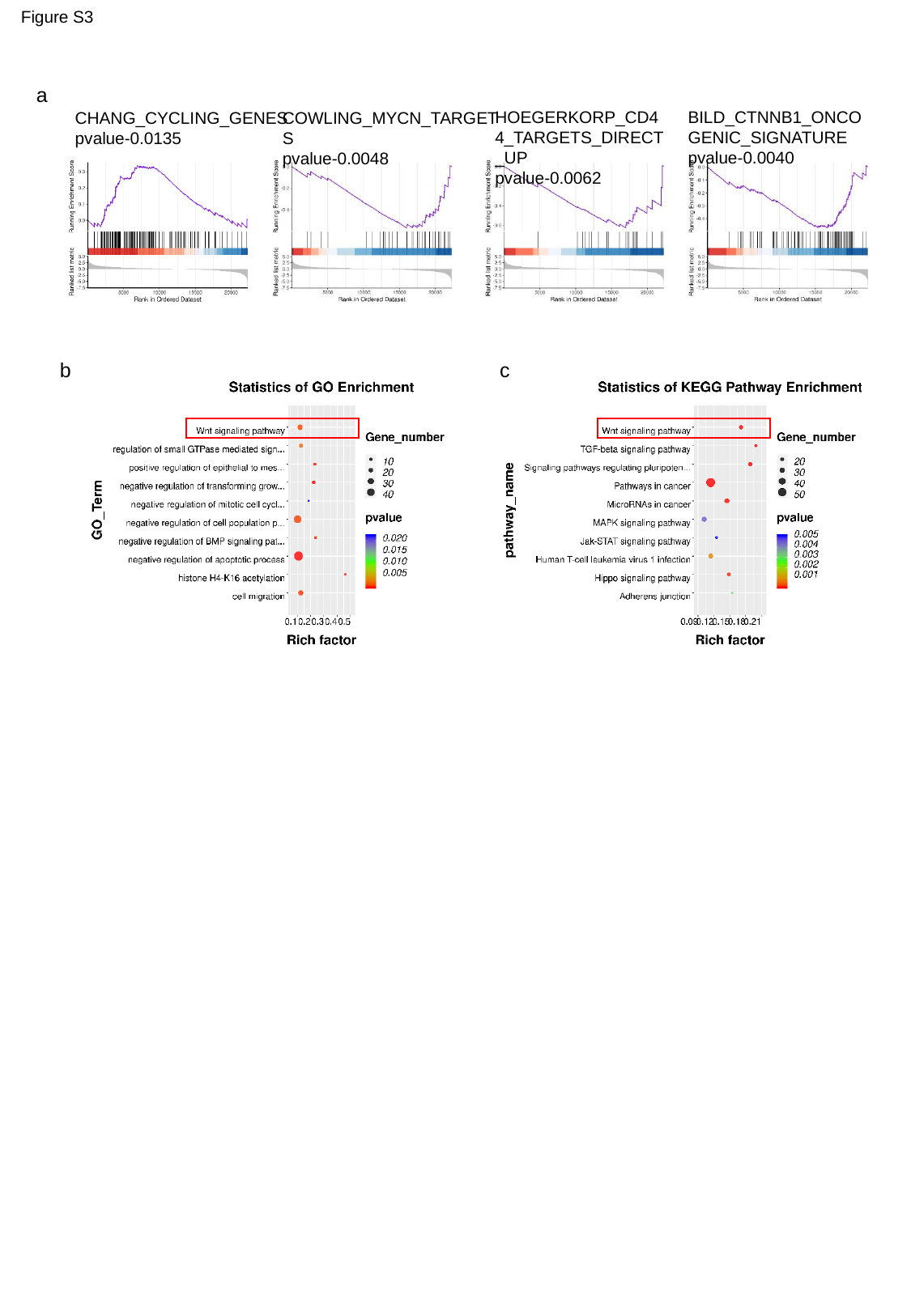

Figure S3
a
HOEGERKORP_CD44_TARGETS_DIRECT_UP
pvalue-0.0062
BILD_CTNNB1_ONCOGENIC_SIGNATURE
pvalue-0.0040
CHANG_CYCLING_GENES
pvalue-0.0135
COWLING_MYCN_TARGETS
pvalue-0.0048
b
c
