# Supplementary material for: RNA m6A reader YTHDF2 facilitates lung adenocarcinoma cell proliferation and metastasis by targeting the AXIN1/Wnt/β-catenin signaling
Source: Cell Death Dis. 2021 May 13;12(5):479. doi: 10.1038/s41419-021-03763-z (PMC8116339; doi:10.1038/s41419-021-03763-z)
Supplement: Supplementary file 3 — Table s1,s2 [file 41419_2021_3763_MOESM3_ESM.docx]

**Table S1:** **Correlation analysis for clinicopathologic variables in YTHDF2 expression among 513 lung adenocarcinoma patients in TCGA database**

| Characteristic | low YTHDF2  n (%) | high YTHDF2  n (%) | *P* value |
| --- | --- | --- | --- |
| Total | 256 (49.9) | 257 (50.1) |  |
| Age, years |  |  | 0.526 |
| ≤ 60 | 81 (15.8) | 81 (15.8) |  |
| > 60 | 175 (34.1) | 176 (34.3) |  |
| Gender |  |  | 0.312 |
| Male | 115 (22.4) | 122 (23.8) |  |
| Female | 141 (27.5) | 135 (26.3) |  |
| Clinical stage |  |  | 0.295 |
| Ⅰ-Ⅱ | 195 (38.0) | 201 (39.2) |  |
| Ⅲ-Ⅳ | 58 (11.3) | 52 (10.1) |  |
| Tumor invasion depth |  |  | 0.809 |
| T1 | 87 (17.0) | 83 (16.2) |  |
| T2 | 135 (26.3) | 144 (28.1) |  |
| T3 | 25(4.9) | 20(3.9) |  |
| T4 | 9(1.7) | 10(1.9) |  |
| Lymph node metastasis |  |  | 0.663 |
| N0 | 166 (32.4) | 174 (33.9) |  |
| N1 | 48 (9.4) | 48 (9.4) |  |
| N2-3 | 42 (8.2) | 35 (6.8) |  |
| Distant metastasis |  |  | 0.350 |
| M0/Mx | 244(47.6) | 242 (47.2) |  |
| M1 | 12 (2.3) | 15 (2.9) |  |

**Table S2: Correlation analysis for clinicopathologic variables in YTHDF2 expression among 131 lung adenocarcinoma patients in CHOICE study**

| Characteristic | low YTHDF2  n (%) | high YTHDF2  n (%) | *P* value |
| --- | --- | --- | --- |
| Total | 65 (49.6) | 66 (50.4) |  |
| Age, years |  |  | 0.457 |
| ≤ 60 | 25 (19.1) | 27 (20.6) |  |
| > 60 | 40 (30.5) | 39(29.8) |  |
| Gender |  |  | 0.218 |
| Male | 48(36.6) | 43 (32.8) |  |
| Female | 16 (12.2) | 21(16.0) |  |
| Clinical stage |  |  | 0.402 |
| Ⅰ-Ⅱ | 46 (35.1) | 49 (37.4) |  |
| Ⅲ-Ⅳ | 19 (14.5) | 17 (13.0) |  |
| Tumor invasion depth |  |  | 0.699 |
| T1 | 14 (10.7) | 18 (13.7) |  |
| T2 | 39 (29.8) | 35 (26.7) |  |
| T3-4 | 12(9.2) | 12(9.2) |  |
| Lymph node metastasis |  |  | 0.500 |
| N0 | 46(35.1) | 45 (34.4) |  |
| N1-2 | 19 (14.5) | 20 (15.3) |  |
| Distant metastasis |  |  | 0.491 |
| M0 | 61(46.6) | 63 (48.1) |  |
| M1 | 4 (3.1) | 3 (2.3) |  |
